# Surgical management strategies and clinical outcomes of cutaneous skeletal hypophosphatemia syndrome: a case series

**DOI:** 10.3389/fped.2025.1742471

**Published:** 2026-01-20

**Authors:** Yi Qiao, Jin Dai, Ting Zhuang, Yicong Liu, Xiuzhi Ren

**Affiliations:** Department of Pediatric Orthopedics, Children’s Hospital of Soochow University, Suzhou, Jiangsu, China

**Keywords:** CSHS, cutaneous skeletal hypophosphatemia syndrome, outcomes, rare disease, surgery

## Abstract

**Objective:**

To evaluate the surgical treatment strategies and clinical efficacy in patients with cutaneous skeletal hypophosphatemia syndrome (CSHS).

**Methods:**

A retrospective analysis was conducted on three cases of CSHS treated at our institution. Clinical data included medical history, physical examination, laboratory tests (hypophosphatemia-related biomarkers and genetic testing), and imaging studies (x-ray and CT). Pre- and postoperative limb deformity correction and functional recovery were assessed.

**Results:**

All three patients presented with multiple skeletal deformities and cutaneous melanocytic nevi. Laboratory tests confirmed persistent hypophosphatemia, while imaging revealed widespread osseous abnormalities and long-bone bowing deformities. Following corrective osteotomy with internal fixation, significant improvement in mechanical alignment was achieved.

**Conclusions:**

Surgical intervention can effectively correct limb deformities, restore biomechanical alignment, and improve function in patients with CSHS. Hence, it represents a critical component of multidisciplinary management.

## Introduction

Cutaneous skeletal hypophosphatemia syndrome (CSHS) is a rare disorder caused by somatic mosaic mutations—most commonly in the *RAS*, *HRAS*, or *NRAS* genes (1, 2). Clinically, it is characterized by skeletal dysplasia (including long bone bowing, scoliosis, and pathological fractures), cutaneous melanocytic or epidermal nevi (typically arranged in a linear distribution and topographically associated with underlying skeletal abnormalities), and fibroblast growth factor 23 (FGF-23)-mediated hypophosphatemia (3, 4). FGF-23 exerts its action by binding to the FGFR-Klotho receptor complex in the kidneys. This binding leads to the downregulation of sodium-phosphate cotransporters (NaPi-IIa/IIc), which causes increased urinary phosphate excretion, and the suppression of 1*α*-hydroxylase activity, which reduces the synthesis of 1,25-dihydroxyvitamin D₃ (5). Collectively, these renal effects result in chronic hypophosphatemia, which underlies the development of bone diseases such as rickets and osteomalacia (6).

Although previous studies have primarily focused on pharmacological interventions—such as phosphate supplementation and active vitamin D therapy—to correct metabolic abnormalities (3, 7–9), systematic research on surgical management of skeletal deformities in CSHS remains limited. To date, very few case reports are available on corrective osteotomies. This study aims to conduct a evaluation of surgical approaches based on detailed clinical data, radiographic findings, and surgical outcomes from three cases of CSHS. Key considerations of the study include the timing of osteotomy, selection of internal fixation techniques, and postoperative rehabilitation protocols, with the aim of providing evidence-based guidance for clinical practice.

## Methods

### Patients

Patients diagnosed with CSHS and treated at Children's Hospital of Soochow University between May 5, 2012, and February 24, 2025 were included. The diagnosis criteria (1) were as follows:

- Persistent hypophosphatemia, defined as serum phosphate levels below the age-specific lower limit of normal (normal range: 1.25–1.93 mmol/L at 6–12 years; 1.15–2.01 mmol/L at 12–15 years);
- clinical manifestation of multiple cutaneous epidermal and/or melanocytic nevi;
- radiographic evidence of skeletal abnormalities, such as long bone bowing or pathological fractures; and
- molecular confirmation of an *HRAS* mutation identified by next-generation sequencing.

All the included patients underwent corrective osteotomy, with comprehensive clinical documentation.

The study protocol was approved by the Institutional Review Board of Children's Hospital of Soochow University (Approval No: 2025CS159). Written informed consent was obtained from the legal guardians of all participants.

### Data collection

Clinical data related to medical history, physical examination, laboratory tests (hypophosphatemia-related biomarkers and genetic testing), and imaging studies (x-ray and CT) were collected. Pre- and postoperative limb deformity correction and functional recovery were assessed using Barthel Index (10) and WeeFIM (11).

#### Surgical treatment

All enrolled pediatric patients exhibited multiple skeletal deformities and underwent femoral, tibial, radius or ulna osteotomies with internal fixation under general anesthesia. The following surgical procedures were performed:

#### Femoral osteotomy

The Sofield–Millar procedure was carried out. First, the patients were positioned laterally. Next, a posterolateral longitudinal incision was made from the greater trochanter to the most distal point of curvature. After layered dissection through the skin and subcutaneous tissues, the vastus lateralis muscle was anterolaterally reflected to expose the femoral periosteum. Following periosteal incision, the deformed bone was visualized, and multiple osteotomies were performed at the sites of maximal bowing. Next, sequential intramedullary reaming was performed. A guide pin was inserted through the greater trochanter, and points of resistance during drilling were identified as osteotomy sites. The bone segments were reshaped as needed. A pre-measured Fassier–Duval (FD) expandable intramedullary nail was then inserted and advanced to the distal femoral epiphysis.

#### Tibial osteotomy

With the patient in a supine position and under the application of a tourniquet, an anteromedial longitudinal incision was made over the most prominently deformed area of the tibial surface. Layered dissection was performed through the skin and subcutaneous tissues, and the tibial periosteum was exposed. Following periosteal incision, the deformed bone was visualized, and multiple osteotomies were performed at the sites of maximal bowing. Sequential intramedullary reaming was performed from both proximal and distal ends. Points of resistance during drilling were identified as osteotomy sites. The total length was measured after performing segmental osteotomy. A Peter–Williams nail was inserted antegradely through the talus and was exited from the plantar surface. It was then advanced retrogradely to the proximal tibial canal and proximally fixed across the tibial physis.

#### Radius and ulna osteotomy

Again, with the patient in a supine position and under the application of a tourniquet, a longitudinal incision was made over the most prominently curved segment of the forearm. A layered dissection through the skin and subcutaneous tissues was performed and the periosteum of the radius and ulna was exposed. Following periosteal incision, the deformed bone was visualized, and multiple osteotomies were performed at the sites of maximal bowing. Next, sequential intramedullary reaming was performed from both proximal and distal ends. However, the radius and ulna exhibited severe deformities, with some parts showing flattened bone-like changes, making it impossible for the drill to pass through smoothly. Multiple osteotomies were performed, and the bone segments were trimmed. After appropriate shortening of the bone segments, the bone length was measured, and elastic intramedullary nails of suitable thicknesses were selected. Next, the intramedullary pinning procedure was performed as follows: for the ulna, the pin was inserted in a retrograde manner, drilled from the proximal end through the skin and then advanced anterogradely to the distal medullary canal; for the radius, the nailing was first drilled anterogradely through the skin and then inserted retrogradely into the proximal medullary canal.

### Follow-up

The follow-up was conducted through outpatient visits. Serial radiographs were obtained at regular intervals to assess bone healing and alignment. Clinical assessments of limb appearance and function were performed during the follow-up. Standardized photographs were taken to document the external limb morphology.

## Results

A total of three male patients (age: 5–12 years) with CSHS were included in this study. The clinical, radiographic, surgical, and functional characteristics of the three cases are summarized in Tables 1, 2.

**Table 1:** Clinical and radiographic characteristics of patients.

Case	Sex	Age at evaluation (Year)	Fracture sites	Diagnosis	Radiographic findings	Clinical presentations	Surgical sites
Case 1	Male	5	Bilateral femurs, bilateral tibiae-fibulae, bilateral ulnae-radii, spine	Hypophosphatemic rickets	Osteoporosis, scoliosis with vertebral compression deformities		Bilateral femurs
		6 (Follow-up)		CSHS	Satisfactory correction of femoral and tibial tibia-fibula curvature with restored mechanical alignment	Assisted walking	Bilateral tibiae-fibulae
		9 (Follow-up)		CSHS	Progressive scoliosis with worsened vertebral compression	Assisted walking	
Case 2	Male	12	Right femoral	Hypophosphatemic rickets			Bilateral femurs, left tibia
		22 (Follow-up)	Right tibia, femoral neck	CSHS			Right tibia
		26 (Follow-up)					Left femur, cannulated screws
Case 3	Male	5	Bilateral femurs, bilateral tibiae-fibulae, right humerus-ulna-radius, spine	Hypophosphatemic rickets	Osteoporosis, multiple bone deformities with bending, ulna-radius cavitation	Limping	Medial femoral and tibial epiphyseal arrest
		7		CSHS	Bowing deformities, abnormal bone texture of the left radius and ulna		Left ulna-radius
		7.2 (Follow-up)			Satisfactory osteotomy healing		

**Table 2:** Functional recovery of three cases.

Case	Barthel Index		WeeFIM	
	Preoperative	Postoperative	Preoperative	Postoperative
Case 1	44	71	83	104
Case 2	64	100	100	126
Case 3	72	85	102	115

### Case 1

A 5-year-old boy presented to our hospital with severe bilateral lower limb bowing deformities and inability to stand independently.

#### Medical history

Lower limb weakness and pain first appeared at 2 years of age and were initially diagnosed locally as “hypophosphatemic rickets”. Despite prolonged treatment, the patient developed severe osteoporosis and progressive skeletal deformities.

#### Physical examination

**Figure 1:**
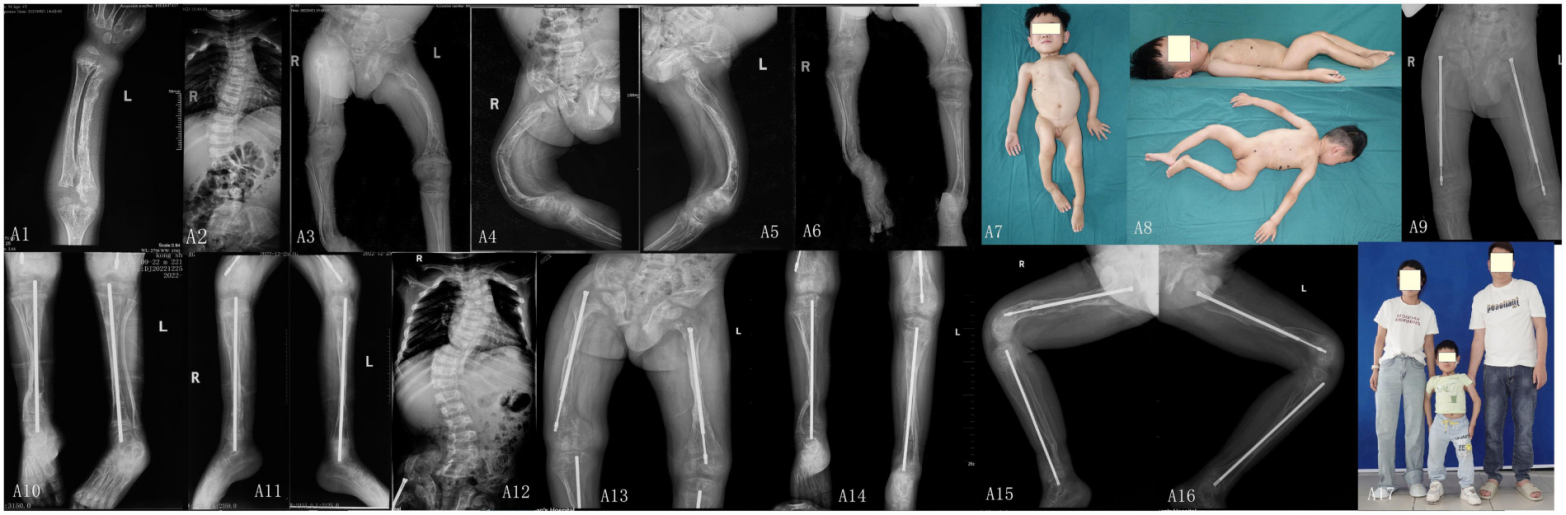
Radiographic and clinical progression of Case 1. **A1**. At age 5, fracture of the left radius and ulna with osteoporosis, showing a cup-shaped deformity of the radius. **A2**. At age 5, scoliosis with vertebral compression deformities. **A3–A6**. Anteroposterior and lateral views of bilateral femurs and tibiae-fibulae show multiple bone abnormalities with deformities, osteoporosis, and obvious bowing deformities of the femurs and tibia-fibula. **A7, A8**. Obvious deformities in the limbs, with significant bowing deformities of the bilateral femurs, tibiae-fibulae, and bilateral forearms. There are also changes in melanocytic nevi on the abdomen, back, neck, and lower jaw. **A9–A11**. At age 6 (1-year post-surgery), x-rays showed satisfactory correction of femoral and tibial tibia-fibula curvature with restored mechanical alignment. Fracture union at osteotomy sites and longitudinal growth of the telescopic Fassier–Duval nails are noted. **A12**. At age 9, progressive scoliosis with worsened vertebral compression. **A13–A16**. At age 9, radiographs at anteroposterior and lateral views show satisfactory fracture union with appropriate elongation of telescopic Fassier–Duval nails and improved bone density. **A17**. Assisted standing with persistent right limb shortening and residual forearm deformities.

Marked bowing deformities of the bilateral lower limbs, forearms, and spine were observed. Multiple scattered cutaneous nevi and melanocytic nevi were distributed across the body. The patient was unable to stand without assistance (Figures 1A7–8). The Barthel Index and WeeFIM scores were 44 and 83, respectively (Table 2).

#### Laboratory findings

Serum phosphorus was markedly reduced at 0.48 mmol/L (normal range: 1.25–1.93 mmol/L at 6–12 years). Genetic testing identified a pathogenic somatic *HRAS* mutation in the lesional epidermis of the melanocytic nevi (Table 3).

**Table 3:** Genetic analysis of three cases.

Patients	Chromosomal coordinate (hg38)	Gene	Nucleotide change	Amino acid change	Zygosity state	Variant classification	Variant origin
Case 1	chr11:533874	*HRAS*	NM_005343.4:c.182A>G	p.Gln61Arg	Mosaic	P	Somatic
Case 2	chr11:533874	*HRAS*	NM_005343.4:c.182A>G	p.Gln61Arg	Mosaic	P	Somatic
Case 3	chr11:534286	*HRAS*	NM_005343.4:c.37G>C	p.Gly13Arg	Mosaic	P	Somatic

#### Imaging

Radiographs showed multiple bowing deformities of the bilateral femurs and tibiae/fibulae, osteoporosis, brush-like metaphyseal changes at the distal radius, and scoliosis with vertebral compression (Figures 1A1–6).

#### Diagnosis

This patient was diagnosed with CSHS.

#### Treatment

Due to marked bilateral lower limb bowing, a staged surgical approach was adopted. Right femoral osteotomy with a telescopic nail was performed first, followed by left femoral osteotomy using a telescopic nail four months later. One month thereafter, bilateral tibial osteotomies with intramedullary nailing were completed.

#### Follow-up

Postoperatively the Barthel Index and WeeFIM scores were 71 and 104, respectively (Table 2). At 1- and 4-year follow-up, implant position remained satisfactory and deformity correction was well maintained. However, scoliosis progressively worsened. The patient continued to require parental assistance for standing, with residual right lower limb shortening and persistent forearm deformities (Figures 1A9–17).

### Case 2

A 12-year-old male patient presented to our hospital with left hip varus deformity, requiring surgical correction.

#### Medical history

The patient first developed weakness in the bilateral lower extremity and gait disturbance at age 3. He was diagnosed with “vitamin D-resistant rickets with hypophosphatemia” at a local hospital. By age 6, he had developed recurrent pathological fractures with malunion, resulting in obvious bowing deformities of both femurs. The patient underwent open reduction and internal fixation with plating for a left femoral fracture at age 7. Sub-plate fracture and screw loosening occurred two years postoperatively. Conservative management of the right femoral fracture at age 11 led to progressive worsening of the deformity.

#### Physical examination

**Figure 2:**
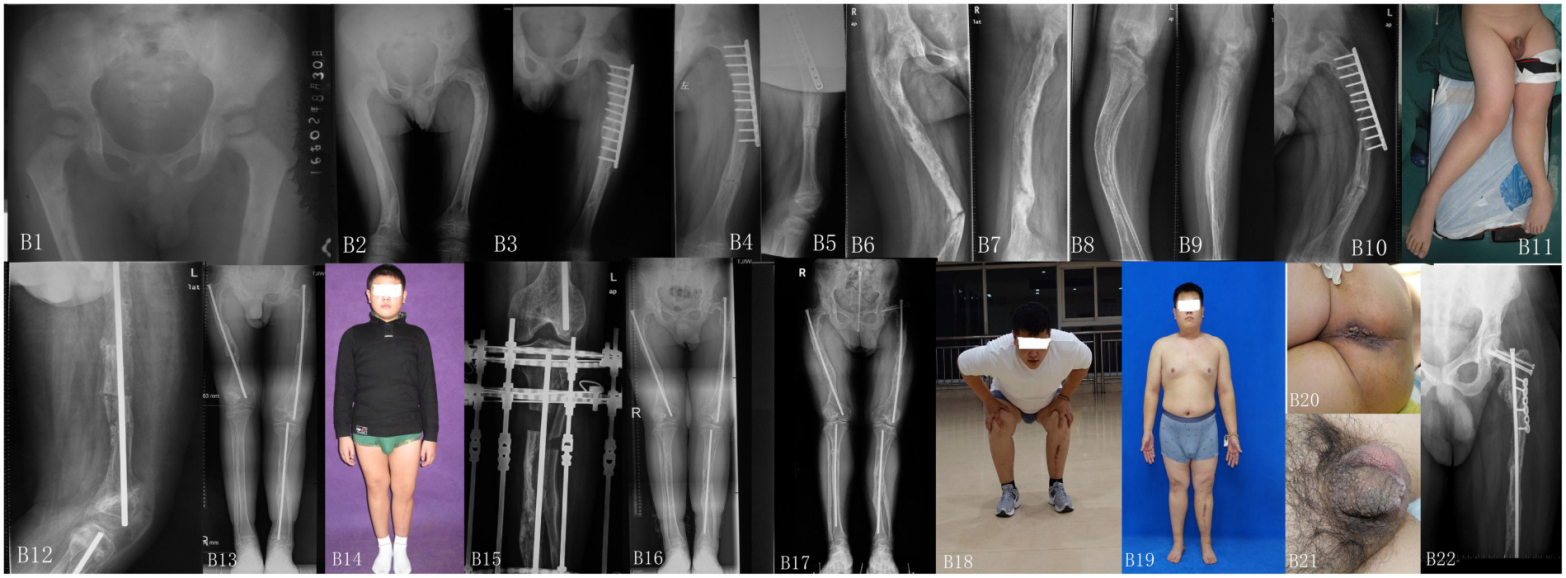
**B1**. At age 2, bilateral proximal femoral fractures with severe osteoporosis. **B2**. At age 6, recurrent fractures with malunion and obvious bowing deformities of the bilateral femurs. **B3**. At age 7, left femoral fracture treated with plate osteosynthesis at an external institution. **B4–B5**. At age 8 (1-year post-surgery), plate dislodgement with distal femoral fracture. **B6**. At age 12, right femoral fracture with distal bowing deformity. **B7**. Left femoral osteoporosis and bowing deformity. **B8–B9**. X-ray of left tibia. **B10**. At age 12, complete dislodgement of the left femoral plate withrecurrent deformity. **B11.** At age 12, severe bilateral lower limb bowing. **B12**. left femoral multilevel osteotomy with P-W intramedullary nailing. Postoperative radiograph showed significant improvement in femoral bowing deformity and restoration of satisfactory mechanical alignment. **B13**. At age 15, radiographs confirmed maintained correction of bilateral femoral and left tibial deformities with stable implants. All osteotomy sites achieved bony union and bone density increased. **B14**. At age 15, asymmetricallimb length and corrected alignment during weight-bearing were noted. **B15**. At age 16, the patient underwent left tibia-fibula osteotomy with external fixation for correcting limb length discrepancy. The lengthening outcome was tisfactory. **B16**. At age 17, the osteoto my site showed good healing. **B17**. At age 22, the patient sustained right tibial fractures and underwent open reducti on and internal fixation (ORIF), with satisfactory postoperative healing. **B18–B19.** At age 25, hip joint mobility was preserved, squatting was slightly limited, the lower limbs were nearly equal in length with no significant deformit ies, and surgical healing was good. **B20–B21**. Numerous epdermal nevi were noted around the anus and penoscrotal regio n during surgery. **B22**. At age 26, the patient underwent left valgus-producing proximal femoral osteotomy with plate.

The patient exhibited equal limb length bilaterally, with limited left hip abduction (20°) (Figures 2B13). The Barthel Index and WeeFIM scores were 64 and 100, respectively (Table 2).

#### Laboratory findings

Laboratory results showed that the calcium level was 2.35 mmol/L (normal range: 2.1–2.8 mmol/L), the phosphorus level was 1.34 mmol/L (normal range: 1.15–2.01 mmol/L at 12–15 years), and the alkaline phosphatase level was 650 U/L. A genetic analysis was conducted, which identified a pathogenic mosaic somatic variant in the *HRAS* gene (chromosomal location chr11:533874) in the lesional epidermis of the epidermal nevi, specifically a missense mutation designated as NM_005343.4:c.182A>G. This variant causes an amino acid substitution of glutamine to arginine at position 61 (p.Gln61Arg) (Table 3).

#### Imaging

The x-ray examination showed right femoral fracture with distal bowing deformity and left femoral osteoporosis with bowing deformity, along with complete plate displacement (Figures 2B5–10).

#### Diagnosis

The patient was diagnosed with CSHS.

#### Treatment

The patient underwent bilateral femoral osteotomies with rush pin fixation. Three months later, the slipped plate was removed, and left femoral osteotomy with Rush pin fixation was performed. Meanwhile, the Barthel Index and WeeFIM scores were 64 and 100, respectively. Eleven months later, right femoral osteotomy with Rush pin fixation was conducted. Postoperatively, both lower limbs were with good restoration of appearance and function (Figures 2B14). He was also treated with oral calcitriol and phosphate supplements to correct the hypophosphatemia.

#### Follow-up

Postoperatively the Barthel Index and WeeFIM scores were 100 and 126, respectively (Table 2). Left tibial lengthening was performed at age 16 using an external fixator for correcting limb-length discrepancy. X-ray imaging demonstrated satisfactory implant position, significant improvement in bony alignment, and complete osteotomy healing, with restored lower limb symmetry and functional standing posture (Figures 2B13,14). Additional lengthening procedures were required at ages 16 (left tibia/fibula) and 17, with successful consolidation (Figures 2B15,16). However, the clinical course was complicated by recurrent fractures at age 22 involving the right tibia (which was treated with intramedullary fixation) and femoral neck (managed by cannulated screw fixation) (Figures 2B17). Most recent evaluation at ages 25 and 26 showed preserved hip joint mobility (with mild squatting limitation), maintenance of limb length equality, absence of significant angular deformities, and satisfactory postoperative healing of all previous interventions (Figures 2B18,19,22). Multiple scattered epidermal nevi were observed in the perianal and penoscrotal regions during the surgery (Figures 2B20,21). At 25 years, the patient had a calcium level of 2.28 mmol/L, a phosphorus level of 0.73 mmol/L, and alkaline phosphatase of 123.4 U/L. Meanwhile, the parathyroid hormone level was 126 pg/mL and the vitamin D (25-OH) level was 36.61 mmol/L. PINP (102 ng/mL) and β-CTX (0.49 ng/mL) were exclusively reported.

### Case 3

A 7-year-old boy presented with a prominent left forearm bowing deformity and hypophosphatemia.

#### Medical history

**Figure 3:**
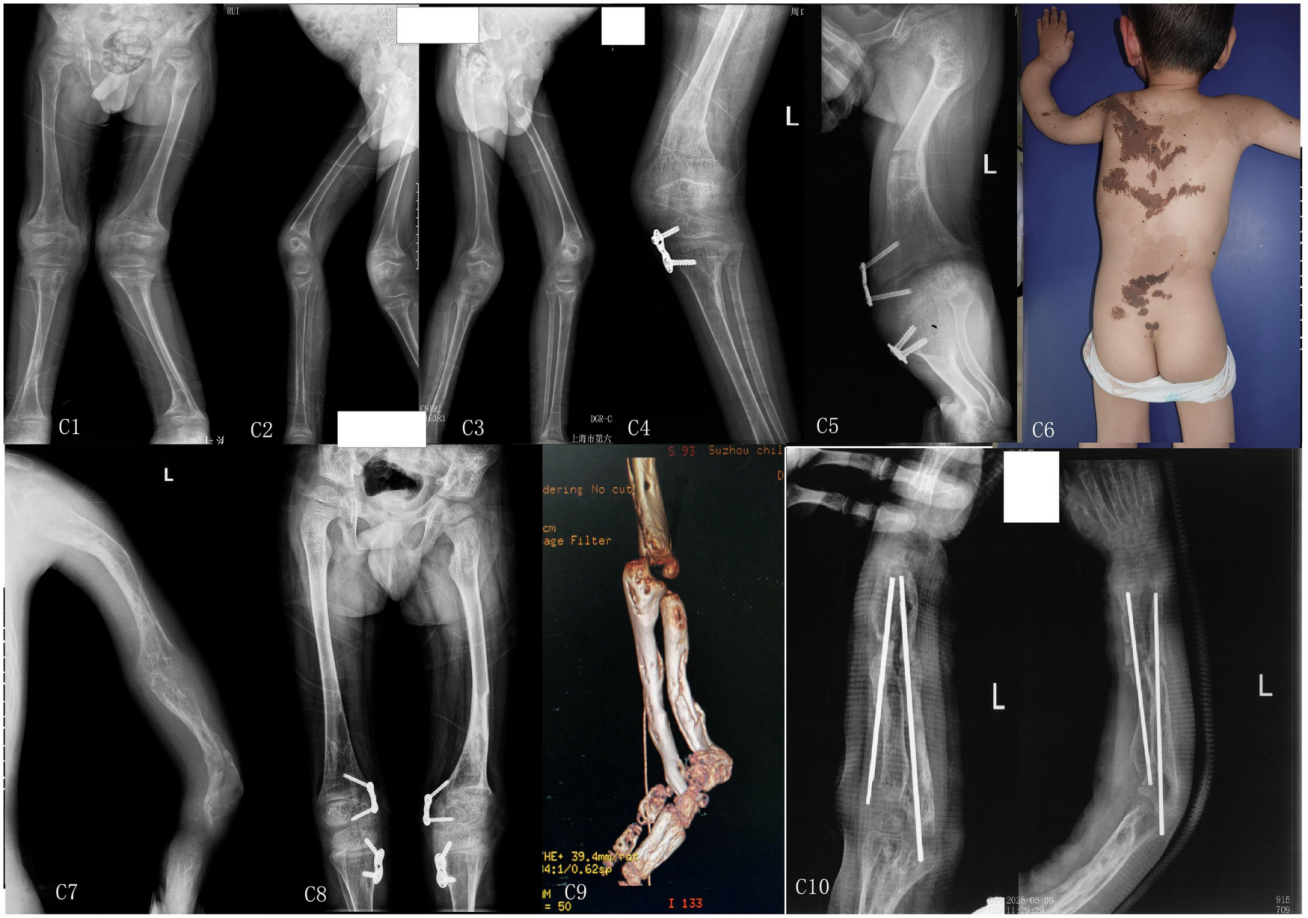
Radiographic and clinical progression of Case 3. **C1–C3**. A 5-year-old male patient presented with anteroposterior and lateral radiographs of the bilateral lower limbs, which revealed marked valgus deformity of the left knee, osteoporosis in both lower limbs, and abnormal fractures with deformities. **C4**. The patient had a left distal femoral fracture. The medial growth plate of the left tibia exhibited signs of growth arrest. **C5**. At age 5, the patient underwent medial femoral and tibial epiphyseal arrest at an external hospital, with a midshaft fracture of the left femur. **C6**. At age 7, the patient exhibited obvious bowing deformity of the left forearm and multiple melanocytic nevi on the back and buttocks. **C7–C9**. x-rays of the left humerus, ulna, and radius showed bone abnormalities, with distal left radius bowing deformity, as well as bilateral femoral bone abnormalities with deformity. CT scans revealed ulnar and radial bone abnormalities, with distal radius bowing accompanied by cavitation. **C10**. Two months following multiple osteotomies and intramedullary nailing of both the radius and ulna, follow-up x-rays demonstrated correction of the radial and ulnar deformities, satisfactory forearm alignment, and successful healing at the osteotomy sites.

At age 5, he underwent medial hemiepiphysiodesis (eight-plate fixation) of the tibia for genu valgum at a local hospital (Figures 3C1–5). Four months later, he sustained a left distal femoral fracture treated conservatively. A recurrent femoral fracture occurred one year afterward.

#### Physical examination

Deformities of all four limbs were noted. Multiple melanocytic and cutaneous nevi were present over the body. A marked left forearm bowing deformity was observed (Figures 1C6). The Barthel Index and WeeFIM scores were 72 and 102, respectively (Table 2).

#### Laboratory findings

Serum phosphorus was 1.14 mmol/L (normal range: 1.25–1.93 mmol/L at 6–12 years). Genetic testing confirmed a somatic *HRAS* mutation in the lesional epidermis of the melanocytic nevi (Table 3).

#### Imaging

Radiographs and CT scans demonstrated bowing deformities and abnormal bone texture of the left radius and ulna, with a cystic-like lesion at the distal radius. Evidence of old fractures and deformities was also found in both femurs (Figures 3C7–9).

#### Diagnosis

The patient was diagnosed with CSHS.

#### Treatment

To improve limb appearance and function, the patient underwent multi-level corrective osteotomies of the left radius and ulna with intramedullary nailing.

#### Follow-up

Two months postoperatively, radiographs showed good restoration of forearm alignment and satisfactory osteotomy healing (Figures 3C10). Three months postoperatively, the Barthel Index and WeeFIM scores were 85 and 115, respectively (Table 2). Functional recovery of the left upper limb was achieved with rehabilitation.

## Discussion

This study included three cases of CSHS. All three patients were admitted owing to multiple skeletal deformities, which were accompanied by widespread melanocytic nevi. Radiographic and CT examinations revealed multiple osseous changes in the affected limbs, with significant visible deformities. Surgical intervention was performed using osteotomy and internal fixation to correct long bone deformities and restore mechanical alignment. Postoperative outcomes demonstrated notable correction of the affected limbs and satisfactory functional recovery. To our knowledge, this is the first study to focus on the surgical management of skeletal deformities in CSHS and to evaluate the operative approach.

CSHS is a disorder characterized by hypophosphatemia, skeletal dysplasia, and melanocytic nevi (12–14). Approximately 20% of patients with CSHS exhibit hemibody asymmetry (3), likely attributable to skeletal deformities and/or abnormal development of the growth plate in the affected limbs (15). Scoliosis appears to be a prevalent feature, as it was reported in up to 40% of cases with CSHS (6). Radiographic examinations typically demonstrate marked osteomalacia accompanied by severe limb deformities (16, 17). All three cases included in our study presented with multiple melanocytic/cutaneous nevi, osseous abnormalities, and significant limb deformities. Notably, Case 1 manifested concomitant scoliosis, consistent with previous literature reports.

Patients with CSHS exhibit refractory hypophosphatemia because of persistently elevated secretion of FGF-23, leading to osteomalacia and skeletal dysplasia (18). Additionally, they present with localized dysplastic skeletal lesions. These patients may harbor pathogenic variants in the RAS family or other factors that disrupt the regulation of bone mineralization, resulting in defective mineralization and impaired fracture healing (19). Consequently, their skeletal deformities are more complex, significantly increasing the surgical challenges.

In this study, surgical intervention was primarily aimed at correcting dysplastic or rachitic skeletal deformities to restore limb alignment and improve the independence and quality of life in patients with CSHS. The surgical approach follows established techniques commonly used for osteogenesis imperfecta and other brittle bone disorders. Because patients with CSHS have reduced bone density and osteoporosis, conventional eccentric plate fixation is unsuitable. Therefore, it is advisable to select the appropriate fixation method based on the specific circumstances of the patient (20, 21). In our study, Case 2 experienced plate detachment and bilateral femoral osteotomies with rush rod fixation were performed. Multisegment osteotomy and intramedullary fixation are the mainstream surgical approaches for treating osteogenesis imperfecta and osteoporosis, as they can correct deformities, enhance limb strength, and reduce the risk of re-fracture. For patients with open epiphyses and significant growth potential (Case 1 and Case 3), the femur can be treated with Fassier–Duval extendable nails (22). If the instability persists at the fracture site and poses a risk to rotational malalignment, additional plate fixation may be required to control the rotation, prevent postoperative femoral internal or external rotation deformities, and address intraoperative rotational issues. For the tibia, the Peter–Williams intramedullary nail (23) can be used to avoid the insertion of the nail through the knee joint, thereby minimizing interference with knee joint mobility and preserving the flexion–extension function. For the radius and ulna, the choice between elastic intramedullary nails and Kirschner wires depends on the bone quality of the patient. Due to bone deformities, some patients may have flattened radii and ulnae with no discernible medullary cavity. In such cases (e.g., Case 3), a 1.5-mm Kirschner wire can be used instead of an intramedullary nail. The osteotomized segments are then aligned and stabilized with the Kirschner wire to restore the length and mechanical axis of the radius and ulna as much as possible. Rehabilitation is recommended one month postoperatively. If radiographic assessment demonstrates abundant callus formation, the cast may be removed and non–weight-bearing exercises gradually initiated. Subsequently, patients may progress to partial and then full weight-bearing with assisted ambulation as tolerated. It is important to note that surgery is only one part of the overall treatment. Since intramedullary nails cannot alter the inherent fragility of the bone, postoperative long-term pharmacological interventions must be performed, including the use of phosphate supplements, active vitamin D₃, and calcitriol or alfacalcidol therapy, with dosing adjusted according to serum biochemical parameters. This principle is exemplified in Case 2, whose serum phosphorus level was within the normal range (1.34 mmol/L), likely reflecting sustained preadmission phosphate supplementation. This case underscores the importance of long-term pharmacological therapy in achieving biochemical stabilization in CSHS-associated hypophosphatemia.

This study confirms that surgical treatment can effectively correct severe skeletal deformities in patients with CSHS. By employing individualized intramedullary fixation and multisegment osteotomy techniques, limb alignment and function can be restored while breaking the vicious cycle of “deformity–disuse–osteoporosis.” The study emphasizes the need to tailor surgical plans based on the age, bone quality, and growth potential of the patient. These findings provide important insights into the comprehensive management of this rare disease, particularly in improving the mobility, preventing secondary deformities, and enhancing the quality of life—all of which are clinically critical parameters.

However, this study has certain limitations: 1) The sample size was small (only 3 cases), which may limit the generalizability of the conclusions; 2) relatively short follow-up periods, which result in the lack of long-term efficacy data; 3) no systematic evaluation of the impact of different medications on surgical outcomes; and 4) residual joint deformities remained after correction of lower limb alignment, limiting the precise assessment of some joint-related measurements. Future studies should increase the sample size and extend the follow-up period to further validate the long-term effectiveness of the surgical approach.

In conclusion, the skeletal deformities in CSHS require a multidisciplinary treatment strategy. Surgical correction can effectively restore limb alignment. Intramedullary fixation is the preferred technique. Moreover, individualized implementation is essential, taking into account the age, deformity characteristics, and bone quality of the patient.

## Data Availability

The datasets presented in this study can be found in online repositories. The names of the repository/repositories and accession number(s) can be found in the article/Supplementary Material.
